# *EGFR*基因突变非小细胞肺癌中基于治疗药物监测的埃克替尼真实世界研究

**DOI:** 10.3779/j.issn.1009-3419.2025.102.02

**Published:** 2025-01-20

**Authors:** Sen HAN, Lan MI, Jian FANG, Xu MA

**Affiliations:** 100142 北京，北京大学肿瘤医院暨北京市肿瘤防治研究所•恶性肿瘤发病机制及转化研究教育部重点实验室; Key Laboratory of Carcinogenesis and Translational Research (Ministry of Education), Peking University Cancer Hospital and Institute, Beijing 100142, China

**Keywords:** 埃克替尼, 肺肿瘤, *EGFR*突变, 不良反应, 治疗药物监测, Icotinib, Lung neoplasms, *EGFR* mutation, Adverse reactions, Therapeutic drug monitoring

## Abstract

**背景与目的:**

真实世界中，埃克替尼（Icotinib）治疗携带表皮生长因子受体（epidermal growth factor receptor, *EGFR*）基因突变的非小细胞肺癌（non-small cell lung cancer, NSCLC）的血浆药物浓度范围尚不明确，药物浓度与其疗效及不良事件之间可能存在关联。本研究通过治疗药物监测（therapeutic drug monitoring, TDM），分析NSCLC靶向治疗中埃克替尼的药物暴露情况，研究埃克替尼的血浆药物浓度与其治疗效果和安全性之间的关系。

**方法:**

前瞻性收集2022年4月至2024年7月在北京大学肿瘤医院接受埃克替尼治疗的伴有*EGFR*敏感突变的NSCLC患者的血液样本，检测埃克替尼的血浆谷浓度，并结合患者的临床病历资料，进一步探究药物浓度与疗效及其毒副作用之间的关系。

**结果:**

22例接受埃克替尼治疗的患者接受了TDM，但其中1例因停药时间过长药物浓度未检测出。其余21例患者，每人抽血1-7次，共获得32份血浆药物浓度数据。埃克替尼药物浓度为126.9-2317.1 ng/mL。21例患者中女性18例（85.7%），男性3例（14.3%），年龄44-85岁。病理类型均为肺腺癌。除5例接受术后辅助治疗外，接受埃克替尼治疗的16例疗效可评价患者的客观缓解率为43.8%（7/16），疾病控制率为100.0%（16/16）。21例患者埃克替尼的中位药物浓度为805.5 ng/mL。疗效评价为部分缓解的患者和病情稳定者相比，其中位药物浓度分别为497.2和1195.5 ng/mL（*P*=0.017）。治疗中未发生不良反应的患者和发生过不良反应者，中位药物浓度分别为997.0和828.6 ng/mL（*P*=0.538）。

**结论:**

埃克替尼在治疗携带*EGFR*基因突变的NSCLC中表现出较好的疗效且具有可耐受的毒性。埃克替尼的血浆药物浓度与其治疗效果之间具有一定的负性相关，而与安全性无明显的关联。

## Abstract (en)

**Background and objective:**

In the real world, the plasma drug concentration range of Icotinib treated with epidermal growth factor receptor (*EGFR*) gene mutant non-small cell lung cancer (NSCLC) is not yet clear, and there may be a correlation between drug concentration and its efficacy, as well as adverse reactions. This study conducted therapeutic drug monitoring (TDM) of Icotinib. The aim of this study was to analyze the drug exposure of Icotinib in targeted therapy for NSCLC, and to investigate the relationship between Icotinib drug concentration and its efficacy and safety.

**Methods:**

Prospective blood samples were collected from NSCLC patients with *EGFR*-sensitive mutations who received treatment with Icotinib in Peking University Cancer Hospital from April 2022 to July 2024. The drug trough concentration of Icotinib in plasma was detected, and the correlation between drug concentration and efficacy, as well as the toxic side effects, were further analyzed based on the patient's clinical medical records.

**Results:**

22 patients who were treated with Icotinib underwent TDM, but one of them did not acquire the data due to prolonged discontinuation. The remaining 21 patients, each with 1-7 blood draws, obtained a total of 32 plasma drug concentration data. The drug concentration of icotinib is a range of 126.9-2317.1 ng/mL. Among the 21 patients, 18 cases were female (85.7%), and 3 cases were male (14.3%), with an age range of 44-85 years old. The pathological types are all lung adenocarcinoma. Except for 5 patients receiving postoperative adjuvant therapy, 16 patients had assessable tumors. The objective response rate was 43.8% (7/16), and the disease control rate reached 100.0% (16/16). The median value of drug concentration is 805.5 ng/mL among those 21 patients. Compared with the patients who achieved stable disease, the median value of drug concentrations of Icotinib in patients who achieved partial response were 497.2 and 1195.5 ng/mL, respectively (*P*=0.017). The median value of drug concentrations for patients who did not experience adverse reactions during treatment and those who experienced adverse reactions were 997.0 and 828.6 ng/mL, respectively (*P*=0.538).

**Conclusion:**

Icotinib demonstrates good therapeutic effect and tolerable toxicity on the *EGFR* gene mutant NSCLC. There is a certain negative correlation between the plasma drug concentration of Icotinib and its efficacy, while there seems no significant correlation with safety.

表皮生长因子受体（epidermal growth factor receptor, *EGFR*）基因是非小细胞肺癌（non-small cell lung cancer, NSCLC）中最常见的肿瘤驱动基因^[1]^。近十几年来，国内已批准并且上市了多种适用于*EGFR*突变NSCLC的靶向药物，即小分子酪氨酸激酶抑制剂（tyrosine kinase inhibitors, TKIs），其中包括第一代的埃克替尼（Icotinib）、吉非替尼（Gefitinib）、厄洛替尼（Erlotinib），第二代的阿法替尼（Afatinib），第三代的伏美替尼（Furmonertinib）、阿美替尼（Almonertinib）、奥希替尼（Osimertinib）和贝福替尼（Befotertinib）等^[2]^。埃克替尼是中国自主研发的首个第一代EGFR-TKIs，在与吉非替尼针对晚期*EGFR*敏感突变NSCLC一线治疗中通过头对头的比较确立了其作为一线治疗药物的适应证^[3]^。另外，在II-IIIA期携带*EGFR*敏感突变NSCLC术后辅助治疗的研究中，与常规术后辅助化疗相比，埃克替尼也显著延长了患者的无疾病生存期（disease free survival, DFS）^[4]^，因此，埃克替尼也获得了术后辅助靶向治疗的适应证。

目前临床常用的靶向药物，初始剂量均为统一的固定剂量，但实际上不同患者的个体间和个体内药代动力学（pharmacokinetics, PK）变异率可能较大。治疗药物监测（therapeutic drug monitoring, TDM）通过测定患者体内的药物水平、药理标志物或疗效指标，利用药代动力学理论，并依据药物的治疗窗，以患者疗效和安全性为最终观察目标，旨在制定符合患者个体需求的给药方案。在肿瘤治疗领域，尤其靶向药物的TDM目前发展相对缓慢^[5]^。尽管埃克替尼的标准用药量为125 mg *tid*，但是随着临床研究的深入开展，埃克替尼可能存在更优的给药剂量。例如，在INCREASE研究中，在*EGFR*外显子21 L858R突变的患者中加倍量使用埃克替尼（250 mg *tid*）时，展现了更高的客观缓解率（objective response rate, ORR）和更长的无进展生存期（progression-free survival, PFS），同时不良反应可耐受^[6]^。

在真实世界中，目前尚无埃克替尼治疗药物检测的文献报道，因此埃克替尼药物浓度与其治疗效果、安全性之间的相关性尚不清楚。本研究通过单中心的前瞻性队列研究，收集了携带*EGFR*敏感突变NSCLC患者接受埃克替尼治疗的相关数据，深入探究埃克替尼的药物浓度与其治疗效果和不良反应之间的相关性。

## 1 资料与方法

### 1.1 临床资料

前瞻性收集2022年4月至2024年7月于北京大学肿瘤医院接受埃克替尼治疗的NSCLC患者。纳入标准：（1）通过病理学确诊的NSCLC；（2）通过组织学或血液标本检测到*EGFR*基因突变，包括常见的外显子19缺失突变和外显子21 L858R突变，罕见的非耐药突变L861Q、G719X和S768I也可纳入；（3）临床分期诊断明确，其中局部晚期和转移性肺癌定义为III或IV期；（4）接受埃克替尼治疗，包括但不限于术后辅助治疗和晚期一线治疗。排除标准：（1）存在*EGFR*基因突变以外的肺癌驱动基因；（2）存在*EGFR*耐药基因突变，如*EGFR*外显子20插入突变；（3）存在严重的心脏、肝肾功能障碍等导致不能接受抗肿瘤治疗；（4）存在严重症状的活动性脑转移或脑膜转移；（5）临床病历资料严重缺失。

本研究为一项前瞻性观察研究，不对患者的用药治疗进行任何干预。所有程序符合北京大学肿瘤医院伦理委员会的标准，并且已获得患者及其家属的知情同意。

### 1.2 方法

#### 1.2.1 研究设计

给予符合纳入标准的患者埃克替尼治疗，直到出现疾病进展（progressive disease, PD）、死亡或毒副作用不可耐受（以最先发生的情况为准），每个疗程21 d。主要观察的指标是总生存期（overall survival, OS）、PFS、ORR、疾病控制率（disease control rate, DCR）和治疗相关不良事件。

埃克替尼的TDM在埃克替尼用药至少1周后采集患者的静脉血液样本，开展药代动力学检测和分析。采血时间点为空腹或者服药前1 h，测得的药物浓度为谷浓度。

#### 1.2.2 血药浓度检测

（1）主要仪器：液相色谱串联质谱检测系统（AB SCIEX Triple Quad^TM^ 4500MD，上海爱博才思分析仪器贸易有限公司，国械注进：20172401554），试剂盒：抗肿瘤药物样本萃取液（浙江迪赛思诊断技术有限公司，浙杭械备20210131号）。（2）试剂：色谱柱：编号KLP-SP2-004（浙江迪赛思诊断技术有限公司）；甲醇（色谱级，Merck）、甲酸（LC-MS级，Thermo）、乙酸铵（LC-MS级，Sigma）、异丙醇（色谱级，Merck）、水（屈臣氏）。（3）样本采集：规律服药11 d后（达稳态），采集患者早晨首次服药前（谷浓度C_last_）静脉全血3.0 mL至真空分离胶采血管中，在4 ^o^C条件下以2500 rpm离心5 min，取血浆于-20 ^o^C冰箱中保存，用于测定患者血药浓度。（4）血浆样本处理：50 µL血浆样本被精密吸取于1.5 mL塑料离心管中，加入200 µL含内标的沉淀剂，涡旋混匀1 min，在4 ^o^C条件下，以13,000 rpm离心5 min后，取上清液20 µL加500 µL 50%甲醇溶液稀释，涡旋混匀1 min，取100 µL至进样瓶，待进样分析。

#### 1.2.3 疗效评估

肿瘤评估于基线、治疗开始后每6-8周进行1次，直至出现PD。疗效评估依据实体瘤疗效评价标准（Response Evaluation Criteria in Solid Tumors, RECIST）1.1版进行分类，包括完全缓解（complete response, CR）、部分缓解（partial response, PR）、疾病稳定（stable disease, SD）和PD。PFS定义为从治疗开始到肿瘤进展（如原发病灶转移、发现新的病灶、肿瘤增长等）或任何原因导致死亡的时间。OS定义为从治疗开始至患者死亡的时间。不良反应的评估，根据美国国立癌症研究所制定的药物毒性评价标准（National Cancer Institute-Common Terminology Criteria for Adverse Event, NCI-CTCAE）4.0版进行。

#### 1.2.4 患者随访

对因毒性反应、症状恶化或临床病情进展而停止治疗，或开始接受后续抗癌治疗的患者进行随访，直至确认其出现PD。随访时间截止到2024年9月30日。

### 1.3 统计学分析

采用SPSS 24.0软件进行统计学分析，计数资料使用频数、率以及构成比进行统计描述。计量资料使用均数（正态分布）或中位数（非正态分布）进行描述分析。不同组间药物浓度的比较采用秩和检验。*P*<0.05为有统计学差异。

## 2 结果

### 2.1 基线资料

22例患者进入该前瞻性队列研究，完成至少1次采血和药物浓度检测。其中1例患者因采血时停药时间过长（>7 d），埃克替尼的药物浓度未检测出。其余21例患者，每人抽血1-7次，共获得32例血浆药物浓度数据。本研究以这21例有完整数据的患者作为主要分析对象。男性3例（14.3%），女性18例（85.7%），年龄44-85岁。病理类型均为肺腺癌。肿瘤分期：I期1例（4.8%），II期1例（4.8%），III期8例（38.1%），IV期11例（52.4%）。*EGFR*突变类型：外显子19缺失突变10例（47.6%），外显子21 L858R突变10例（47.6%）、L861Q突变1例（4.8%）。埃克替尼作为晚期一线治疗12例（57.1%），二线及以上治疗1例（4.8%），术后辅助治疗5例（23.8%），新辅助治疗3例（14.3%）。

### 2.2 埃克替尼的治疗效果

21例患者中除5例接受术后辅助治疗患者外，共有16例可评价疗效的患者。这些患者均完成影像学复查并评估了疗效。在治疗过程中达到的最佳治疗效果为7例患者达到PR，9例患者保持SD，未观察到CR和PD的患者，ORR为43.8%（7/16），DCR达到100.0%（16/16）。

### 2.3 治疗相关的不良反应

21例患者中，11例患者未出现明显不良反应，而10例患者发生了与埃克替尼治疗相关的不良事件，包括：肝功能异常5例、皮疹及白细胞减少各2例、腹泻、腹痛、发热、肌酸激酶升高、血小板减少、乏力、脱发各1例。其中大部分为1级，只有1例患者因不良反应终止治疗，该患者反复出现血小板减少2度，肝功能异常1度。具体不良反应见表1。

**表1:** 埃克替尼相关不良反应

Adverse events	Grade	n	Percentage
Liver dysfunction	1	5	23.8%
Rash	1	2	9.5%
Decrease of white blood cell	1	2	9.5%
Diarrhea	1	1	4.8%
Abdominal pain	2	1	4.8%
Fever	2	1	4.8%
Increase of creatine kinase	1	1	4.8%
Decrease of platelet	2	1	4.8%
Fatigue	2	1	4.8%
Alppecia	1	1	4.8%

Multiple adverse reactions may simultaneously occur in one patient.

### 2.4 埃克替尼的药物浓度

21例患者中，每人抽血1-7次，共获得32份血浆药物浓度数据（图1）。32份血样中埃克替尼的药物浓度为126.9-2317.1 ng/mL，中位数为895.0 ng/mL。当患者抽血大于1次时，将采取平均值，作为该患者的血浆药物浓度。21例患者的埃克替尼药物浓度为126.9-2317.1 ng/mL，中位数为805.5 ng/mL。其中，埃克替尼作为术后辅助治疗的5例患者的中位药物浓度为1002.6 ng/mL，其他患者的中位药物浓度为890.0 ng/mL，但二者之间无明显统计学差异（*P*=0.726）。

**图1:**
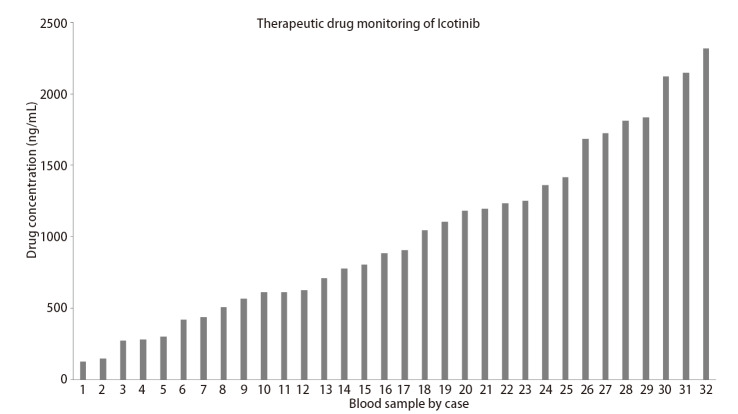
埃克替尼血浆药物浓度监测数据

本研究中有19例患者应用常规固定剂量（125 mg *tid*），2例患者采用加倍剂量（250 mg *tid*），接受加倍剂量的患者均携带*EGFR*外显子21 L858R突变。接受常规剂量的患者的埃克替尼平均药物浓度为956.6 ng/mL，接受加倍剂量的患者为538.9 ng/mL。

### 2.5 药物浓度与疗效之间的关系

可评价疗效的患者16例，其中7例PR，9例SD。评效达到PR患者的中位药物浓度为497.2 ng/mL，评效为SD患者的中位药物浓度为1195.5 ng/mL，二者存在统计学差异（*P*=0.017）。

### 2.6 药物浓度与不良反应的关系

21例患者中未发生不良反应的患者血浆中的埃克替尼中位药物浓度为997.0 ng/mL；出现不良反应患者的中位药物浓度为828.6 ng/mL，二者之间无明显统计学差异（*P*=0.538）。

发生2级以上不良反应患者3例，其埃克替尼中位药物浓度为647.5 ng/mL；未发生不良反应或仅发生1级不良反应患者18例，中位药物浓度为961.7 ng/mL，二者之间无明显统计学差异（*P*=0.419）。

### 2.7 随访

截至2024年9月30日，21例患者均未失访。其中8例患者因PD或者埃克替尼导致的不良反应停止治疗，其余13例患者继续接受埃克替尼治疗。

## 3 讨论

埃克替尼已获批用于一线治疗携带*EGFR*突变的晚期NSCLC患者，同时也适用于II-IIIA期NSCLC的术后辅助治疗。CONVINCE研究^[7]^在具有*EGFR*基因突变的晚期NSCLC患者中对比了一线治疗中埃克替尼单药与培美曲塞+顺铂联合培美曲塞维持治疗的疗效与安全性。研究发现埃克替尼组中位PFS为11.2个月，化疗组为7.9个月（HR=0.61, *P*=0.006）。该研究奠定了埃克替尼在晚期一线治疗中的地位。此外，一项随机、双盲、平行对照III期研究（ICOGEN^[3]^）比较了埃克替尼与吉非替尼治疗既往接受过1-2次化疗的局部晚期或转移的NSCLC患者（400例）中的有效性和安全性，结果显示埃克替尼的PFS不劣于吉非替尼（HR=0.84，95%CI: 0.67-1.05；PFS: 4.6 *vs* 3.4个月；*P*=0.13）。与吉非替尼相比，埃克替尼的药物相关不良反应较少（61% *vs* 70%, *P*=0.046），尤其是与药物相关的腹泻发生率更低（19% *vs* 28%, *P*=0.033）。ICOGEN研究中报道埃克替尼的ORR为27.6%，DCR为75.4%，略低于本研究中的43.8%（7/16）和100.0%（16/16），考虑主要原因是本研究中大部分患者为一线治疗的患者，还有新辅助治疗患者，后线治疗患者少。本研究中不良反应的情况与既往研究相当，说明埃克替尼的总体安全性较好。

本研究关注的重点是埃克替尼的血浆中稳态谷浓度。由于埃克替尼的半衰期较短，推荐剂量为125 mg *tid*，连续口服11 d后，药物浓度可以达到稳态。此外，体外研究^[8]^显示埃克替尼与人蛋白的结合率约为98.5%，其代谢主要依赖CYP2C19和CYP3A4酶。目前，尚无学者明确推荐埃克替尼使用的适宜浓度范围。一项I期临床研究^[9]^纳入了36例NSCLC患者，发现埃克替尼的浓度[曲线下面积（area under the curve, AUC）、血药峰浓度（C_max_）]与最佳总体反应率没有相关性，与皮肤毒性、腹泻也没有明显的相关性。但另一项纳入30例患者的研究^[10]^表明，治疗达稳态时，较长的达峰时间（T_max_）与更好的DCR显著相关（*P*=0.011），且最终时间点浓度（C_last_）与PFS呈正相关（*P*=0.016）。本研究中，埃克替尼药物浓度的波动范围比较大（126.9-2317.1 ng/mL），说明相同剂量的药物，在不同患者体内的代谢、分布可能存在较大差异。埃克替尼的中位药物浓度为805.5 ng/mL，这与既往文献^[8]^报道一致。

既往关于EGFR-TKIs TDM的文献较少，其中Boosman等^[11]^首次报道了真实世界中第三代EGFR-TKIs奥希替尼的药物暴露与疗效之间的关系。该回顾性研究分析了145例携带*EGFR*突变NSCLC患者接受奥希替尼治疗的药物浓度与其PFS之间的关系，结果显示奥希替尼的治疗窗很宽，药物谷浓度以166 μg/L为界值，实际测得的浓度≥166 μg/L的患者PFS明显短于<166 μg/L的患者（9.3 *vs* 13.3个月，*P*=0.03），但是后续的单因素和多因素生存分析均显示药物暴露与其疗效无明显的相关性。这与本研究的结果有一定相似之处，因为本研究中，疗效好的患者药物浓度反而低（暴露量小）。类似的，国外另一项多中心的回顾性研究^[12]^显示，EGFR-TKIs的暴露量与其疗效可能存在负相关。该研究将87例患者奥希替尼的药物谷浓度按照四分位数进行分类，血浆谷浓度的第4个四分位数（Q4），即数值>235 ng/mL的患者中位OS比Q1-Q3组缩短约10个月（12.2 *vs* 22.7个月），但差异没有统计学意义（*P*=0.15）。为了进一步确认这一结果，研究人员在41例接受厄洛替尼治疗的NSCLC患者队列中探索了暴露与生存的关系，结果显示Q4厄洛替尼暴露组（>1728 ng/mL）的中位OS比Q1-Q3组明显缩短（4.8 *vs* 22.8个月，*P*=0.0001）。这些结果表明，高暴露于EGFR抑制剂可能与NSCLC患者的生存率较低有关。虽然本研究采用的疗效指标是ORR，但在一定程度上也反映了这一问题，即较高的暴露量对应的是较差的疗效。但后来日本的一项小规模研究^[13]^报道了不同的结果，该研究以奥希替尼药物浓度211 ng/mL为界值，高暴露组相较低暴露组有着更长的PFS（46.3 *vs* 16.8个月，*P*=0.029）。EGFR-TKIs暴露量与不良反应方面，有研究^[14]^纳入159例接受奥希替尼治疗患者的819个样本进行分析，结果显示奥希替尼的清除率（暴露量）与严重毒性显著相关（HR=0.93, 95%CI: 0.88-0.99）。奥希替尼药物浓度的毒性界限值为259 ng/mL，高暴露组患者药物剂量减少50%，将使严重毒性的风险降低53%。该研究同样显示药物暴露与PFS和OS无关。

目前，国内尚未有关于埃克替尼药物浓度与其疗效和安全性之间关系的研究报道。本研究中，评效达到PR的患者的药物浓度明显低于评效SD的患者（未达到PR），分别为497.2和1195.5 ng/mL（*P*=0.017），该结果在一定程度上反映出较高的药物浓度并不意味着疗效更好。考虑可能的原因是：（1）埃克替尼作为一种高效的EGFR抑制剂可能并不需要很高的药物浓度就能实现显著的疗效。（2）肿瘤组织中药物分布和血浆中药物分布的不一致性，即药物更多地分布于肿瘤组织，导致外周血中埃克替尼的浓度反而低，而因为有更多的药物作用于肿瘤，所以抗肿瘤疗效会更好。对于这一机制的验证，需要对肿瘤组织中药物浓度进行检测。（3）肿瘤微环境的影响，或患者代谢酶活性不同，也可能影响血浆药物浓度与疗效的关系。研究数据显示，埃克替尼在发生过1级及以上不良反应的患者中的中位药物浓度与未发生不良反应患者相似，分别为997.0和828.6 ng/mL，两者无显著差异（*P*=0.538），这意味着在常规剂量下，埃克替尼的药物浓度与不良反应的发生无明显的相关性。另外，因为埃克替尼本身的安全性较好，因此在常规用药剂量下，药物浓度不会对不良反应的发生和严重程度起关键作用。这一点与INCREASE研究^[6]^的结果相符，埃克替尼从125 mg *tid*加量到250 mg *tid*时，不良反应发生率才略有增加。本研究中，埃克替尼加倍剂量的患者，其血浆药物浓度并不高，可能就是因为体内暴露量低，效果一般才加量的，而加量后才能和常规剂量患者的药物浓度相当。这在一定程度上也能反映出基于体内暴露量而调整方案的临床价值。在其他肺癌靶向药物中，我们也发现了类似的结果，例如，赛沃替尼治疗间质上皮细胞转化因子（mesenchymal epithelial transition factor, *MET*）基因突变的NSCLC时，治疗效果较好且不良反应可耐受。在常规用药剂量下，赛沃替尼的血浆药物谷浓度与治疗效果之间可能存在负相关关系，但与不良反应的发生没有明显的关系^[15]^。

本研究在国内首次报道了埃克替尼药物浓度与疗效和安全性的关系，但本身还存在许多不足之处：（1）研究样本量较少，且所有病例均来自北京大学肿瘤医院单中心，可能导致地域局限性和选择偏倚。（2）由于样本量有限，无法进行多因素回归分析以确定影响埃克替尼疗效和安全性的独立风险因素。后续我们将扩大样本量，当病例数达到40例以上时，利用受试者工作曲线，确定药物浓度的阈值，才能够更好地反映药物浓度与疗效和不良反应的关系。（3）本研究患者采集仅为稳态谷浓度PK血，无法计算AUC，因此未能更全面地分析药物浓度与不良反应之间的关系。（4）其主要代谢酶CYP2C19具有基因多态性，在亚洲人群存在较高的变异频率，可能导致个体间暴露差异，本研究未检测其代谢酶表型。

综上，通过单中心临床观察和药物浓度分析表明，埃克替尼在治疗携带*EGFR*突变的NSCLC患者中展示出较高的ORR和DCR，且具有可耐受的不良反应。在常规用药剂量下，埃克替尼的血浆药物浓度可能与疗效具有一定的负相关性，与不良反应无显著的相关性。然而，这些发现需要更多的临床数据来进一步验证。
